# Safety and feasibility analysis of rapid daratumumab infusion in Chinese patients with multiple myeloma

**DOI:** 10.1002/cam4.7347

**Published:** 2024-06-07

**Authors:** Xi‐xi Yin, Yueyun Hu, Yusi Yang, Xinglan Zhang, Li Liu, Xi Cao, Jianwen Chen, Zhongjun Xia, Ye Wang

**Affiliations:** ^1^ Sun Yat‐sen University Cancer Center Guangzhou Guangdong China; ^2^ The Third Affiliated Hospital Sun Yat‐sen University Guangzhou Guangdong China; ^3^ Sun Yat‐sen University School of Nursing Guangzhou Guangdong China

**Keywords:** Chinese patients, daratumumab, infusion‐related reactions, multiple myeloma, rapid infusions, safety, symptoms

## Abstract

**Background:**

With the increasing use of daratumumab (DARA)‐containing regimens for multiple myeloma (MM) patients in China, the standard infusion time of DARA is long, with the potential for infusion‐related reactions (IRRs) and increased hospitalization and use of resources. Shortening the duration of DARA infusion helps to optimize the hospital stay and enhance the patient treatment experience. The current, commonly used 90‐min rapid DARA infusion regimen may not be applicable to Chinese MM patients, and therefore, we explored a new 110‐min rapid DARA infusion regimen aimed at reducing the treatment burden on patients to guarantee therapeutic safety.

**Methods:**

MM inpatients treated with the DARA regimen were divided into two groups according to the number of times the DARA regimen was used: a standard infusion regimen for patients treated with the first two doses of DARA and a 110‐min rapid infusion regimen for patients treated with more than two doses of DARA. Anti‐allergy medications were routinely administered prior to the start of DARA infusion, patient consent, and authorization was obtained for all treatments, and statistical evaluation of the results was conducted via descriptive analyses, one‐way ANOVA and chi‐square tests.

**Results:**

A total of 129 patients were included in this study: 68 in the standard infusion group, with 121 DARA infusions, and 129 in the rapid infusion group (patients who participated in the standard infusion subsequently participated in the rapid infusion), with 738 DARA infusions. The incidence of IRRs was 27.27% (36/121) in the standard infusion group and 1.35% (10/738) in the rapid infusion group, which were significantly different (*p* < 0.001). The incidence of IRRs after rapid infusion in other studies was <6%. The incidence of grade 1 IRRs in the rapid infusion group was 0.81% (6/738), the incidence of grade 2 IRRs was 0.54% (4/738), and there were no IRRs above grade 3; age, sex, and underlying disease had no effect on the choice of infusion method (*p* > 0.05). The mean infusion time after the occurrence of IRRs was also shorter in the rapid infusion group than in the standard infusion group (*F* = 24.781, *p* < 0.001).

**Conclusion:**

The 110‐min rapid infusion DARA regimen is feasible and safe for use in Chinese MM patients.

## 1 INTRODUCTION

Multiple myeloma (MM) is a malignant clonal disease of plasma cells that secrete monoclonal immunoglobulins, often resulting in end‐organ damage, including anemia, hypercalcemia, bone destruction, and renal insufficiency.^1^ The proportion of MM cases among the total number of cancer‐related deaths is relatively low. However, in China, the mortality rate of MM has been increasing rapidly in the last decade.^2^ Although almost all patients may relapse and die from the disease, the emergence of new drugs (such as proteasome inhibitors (PIs) and immunomodulatory drugs (IMiDs)) may prolong survival and improve health‐related quality of life.^1^, ^3^, ^4^

Currently, the most commonly used monoclonal antibody in China is daratumumab (DARA), a human monoclonal antibody that targets CD38 (which is highly expressed on myeloma cells) and possesses direct and indirect antitumor activity, as well as a variety of mechanisms of action, including the induction of apoptosis, immune‐mediated effects, and immunomodulatory functions.^5^, ^6^ The dose and frequency of DARA use are patient specific. Generally, DARA is administered as an intravenous injection. During the initial treatment phase, DARA is usually given once a week for 8 weeks. Thereafter, the frequency of dosing may be reduced to once every 2 weeks for 9–16 weeks and then once a month until the end of the course of treatment.^7^, ^8^ To minimize infusion‐related reactions (IRRs), the median duration of the first infusion of DARA is 7.5 h, with subsequent infusions taking 3.5–4 h.^8^ However, treatments that include the DARA regimen impose a greater burden on patients and healthcare resources due to the need for prolonged infusions.

MM is common in elderly patients who are in poor physical condition and have many comorbidities, and prolonged infusion can easily cause fatigue, anxiety, irritability, and other discomfort. Many studies^9^, ^10^, ^11^, ^12^, ^13^, ^14^, ^15^, ^16^, ^17^ have shown that the majority of DARA IRRs occur during the first and second infusions, with a significantly lower incidence of IRRs occurring during subsequent infusions. If the infusion rate is accelerated and the infusion time is shortened after the second infusion, patient comfort can be improved and patient anxiety can be reduced. There are a variety of rapid DARA infusion regimens available, the more common being the 90‐min rapid infusion regimen. In the 90‐min rapid infusion regimen, 20% of the DARA dose is infused in the first half hour (200 mL/h), and the remaining 80% of the dose is infused in the remaining 1 h (400 mL/h). Barr and colleagues were the first to report that 28 MM patients receiving 90 min of rapid infusion had no infusion reactions, and only one patient experienced a grade 2 hypertension event.^9^ Other studies^10^, ^11^, ^12^, ^13^, ^15^, ^16^ have also concluded that this regimen may be feasible. Bonello et al.^15^ reported that when a 90‐min rapid infusion regimen was used for DARA infusion into 134 MM patients, 7 (5%) developed grade 1 and 2 IRRs.

However, the unanimous consensus is that only the infusion rate can be increased after the second DARA infusion, and the exact timing and rate of the infusion need to be explored. Using a rapid infusion regimen with an infusion time <110 min, Gordan et al.^15^ reported that no IRRs occurred in 147 MM patients. Wang et al.^17^ reported that a 120‐min rapid infusion DARA regimen applied to 60 patients with MM resulted in a grade 1 and 2 IRR in only three patients (5%), which consisted of 20% of the DARA dose occurring within 30 min (200 mL/h) and 80% occurring within 60 min (270 mL/h).

With the increasing frequency of use of DARA‐inclusive regimens in China, existing inpatient resources can no longer meet the demand for medication due to the longer timing of DARA infusions, we aimed to transfer DARA infusion to ambulatory or outpatient settings. Thus, our study was conducted to explore a safe and reliable DARA infusion regimen. In accordance with other studies,^15^, ^17^ we considered older MM patients and the possibility that the faster infusion rate of the 90‐min rapid infusion regimen may cause circulatory problems. Finally, we designed a new DARA infusion regimen and explored its safety and feasibility in Chinese MM patients.

## 2 MATERIALS AND METHODS

### 2.1 Study design and patient selection

**FIGURE 1:**
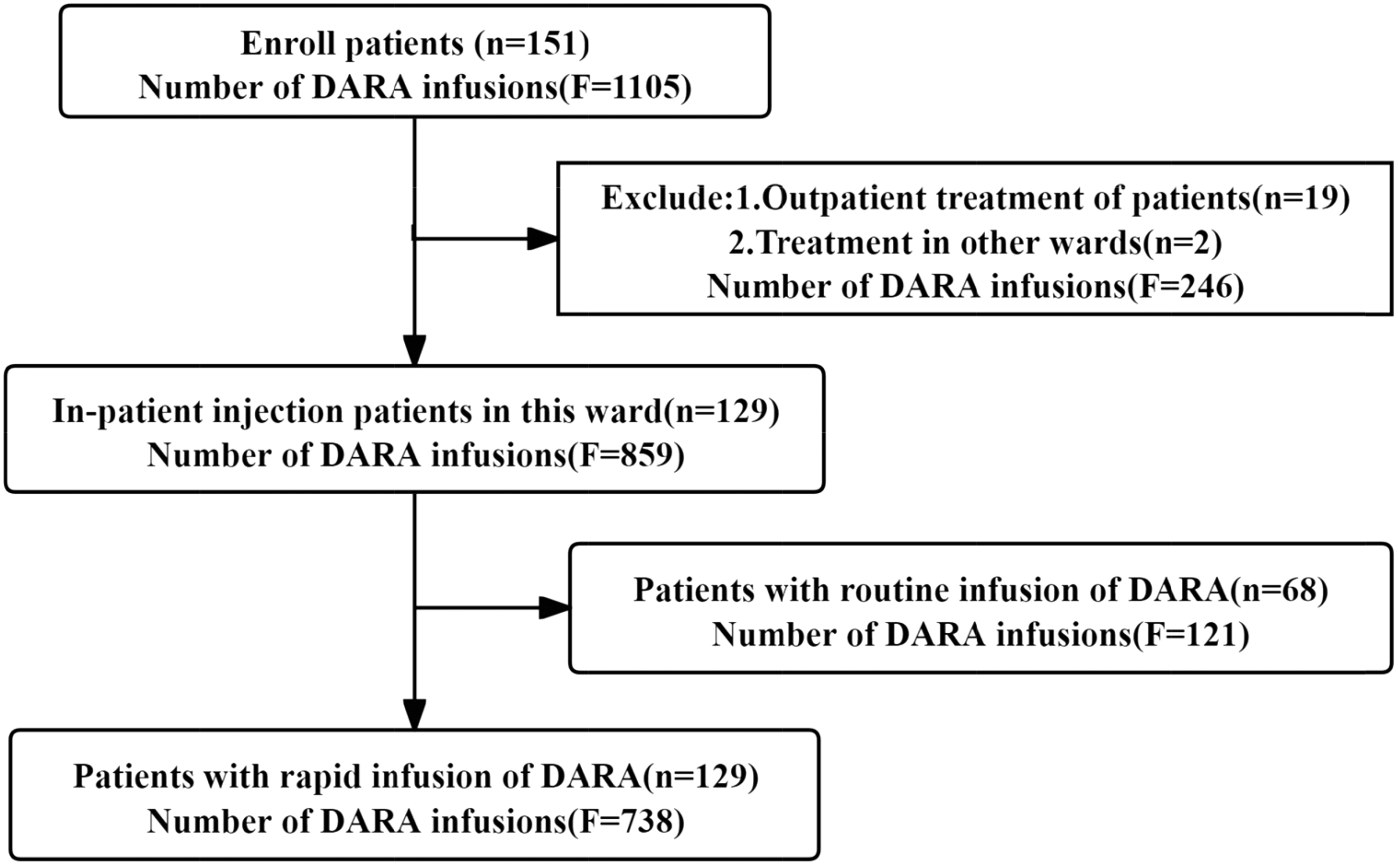
Number of Daratumumab injections and patients. DARA, Daratumumab; F, frequency; n, number.

This was a prospective, single‐center, observational study aiming to explore the safety of rapid, 110‐min DARA infusion in MM patients treated at a Chinese hospital (Sun Yat‐sen University Cancer Centre) after implementation of this regimen in clinical practice. Our study included a total of 151 patients with MM who were treated between April 1, 2023, and February 29, 2024, and who received a total of 1105 DARA injections. The following inclusion criteria were used: (1) had received ≥2 prior DARA infusions; (2) did not have a prior grade 3 or greater IRR; (3) signed a notification; and (4) were inpatients in our department. The exclusion criteria for patients were as follows: (1) had ≥grade 3 IRRs during previous DARA infusions; (2) had comorbidities such as chronic obstructive pulmonary disease, high blood pressure, severe asthma, chronic obstructive pulmonary disease (COPD), or New York Heart Association (NYHA) class III or IV disease; and (3) were injected on an outpatient basis, with the investigator unable to intervene in the method of injection. The final number of patients who participated in our study was 129, with a total of 738 injections, as detailed in Figure 1. The study was approved by an ethics committee (Ratifying body: Ethics Committee of the Sixth Affiliated Hospital of Sun Yat‐sen University, Number 2021ZSLYEC‐394), and all the procedures were performed in accordance with the Declaration of Helsinki.

The 110‐min rapid infusion regimen was as follows: all patients included in the study were dosed according to the standard instructions for the ≤2 infusions of DARA, and the patients could tolerate an infusion rate of 200 mL/h before the rapid infusion could be performed. The infusion rate was increased with the same dosage infusion, with the first 20% of the total volume of the fluid being infused for half an hour at 200 mL/h and the remaining rehydration fluid being infused at a rate of 300 mL/h. The infusion was completed in approximately 80 min, as shown in Table 1. To prevent the occurrence of infusion‐related reactions, anti‐allergic drugs were used before both standard and rapid infusion of DARA, nonsteroidal anti‐inflammatory drugs (such as celecoxib) were added to the anti‐allergic drugs for the first infusion, and antihistamines (such as promethazine hydrochloride) were added in the case of grade 2 or higher IRRs during the infusion. The specific drugs used were as follows: 200 mg of celecoxib, 20 mg of dexamethasone, and 20 mg of diphenhydramine.

**TABLE 1:** Daratumumab comparison of different infusion regimens.

Number of infusions	Infusion rate (mL/h)	Infusion time (h)	Cumulative Infusion(mL)
Standard infusion: First infusion	50	0–1	25
	100	1–2	150
	150	2–3	300
	200	3–6.5	1000
Standard infusion: Second infusion	50	0–1	50
	100	1–2	150
	150	2–3	300
	200	3–4	500
90 min rapid infusion	200	0–0.5	100
	400	0.5–1.5	500
120 min rapid infusion	200	0–0.5	100
	270	0.5–2	500
Our rapid infusion (110 min)	200	0–0.5	100
	300	0.5–1.83	500

Abbreviation: h, hour.

### 2.2 Study measures

Monitoring of adverse reactions: A nurse in our study recorded the occurrence of uncomfortable symptoms, the use of anti‐allergy medication, and the subsequent infusion of DARA. Adverse reactions were evaluated according to the National Cancer Institute (NCI) Common Terminology Criteria for Adverse Events (CTCAE, version 5.0) and were categorized into grades 1–4. If the patients experienced grade 3–4 IRRs, the infusion was immediately terminated, symptomatic treatment was given, and the next infusion was switched to the standard infusion regimen; otherwise, the next cycle of treatment could be continued with the rapid infusion regimen.

Study aims: The aim of this study was to explore the safety and feasibility of 110 min of rapid DARA infusion in Chinese patients with MM according to the incidence, type, and severity of the outcome target IRRs.

### 2.3 Statistical analysis

A descriptive approach was used to summarize demographic characteristics, clinical characteristics and safety data. Count data are described herein as frequencies (percentages), and measurement data are described as medians (ranges) or means (*x*) ± standard deviations (SDs). One‐way analysis of variance (ANOVA) was used to compare the infusion times after the occurrence of IRRs between the standard infusion regimen and rapid infusion regimen. The Chi‐square test (*χ* ^2^) was used to compare the incidence of IRRs, and *p* < 0.05 was considered to indicate a statistically significant difference.

## 3 RESULTS

### 3.1 General information results

In our study, a total of 151 patients were initially enrolled and participated in a total of 1105 DARA injections. The final number of enrolled inpatients was 129, of whom 68 were injected with DARA for the first time. The first two injections of DARA were performed using a conventional infusion protocol with 121 injections (some patients went to the outpatient clinic for injections, and it was not possible to observe complications), and the third injection of DARA was started using a rapid infusion regimen for the third DARA injection. The final 129 patients received rapid infusions for a total of 738 injections, as detailed in Figure 1.

Table 1 shows the durations of different infusion regimens after the second DARA infusion for the same total amount of infusion (500 mL). The time required for regular infusion is 3–4 h, and the time required for rapid infusion is as short as 1.5–2 h. The regimen used in our study was as follows: the first 20% (100 mL) of the infusion was injected at a rate of 200 mph for 0.5 h, and the second 80% (400 mL) was injected at a maximum rate of 300 mph until completion. At a maximum rate of 300 mL/h until completion, the time required was 110 min, and the typical dose for each infusion was calculated as follows: body surface area (m^2^) × 16 mg/kg.

The 129 patients included in this study received a total of 859 injections, and the treatment regimen used is shown in Table 2. The regimen generally consisted of the addition of a nonsteroidal anti‐inflammatory drug (celecoxib 200 mg) before the first administration of DARA, as well as a doubling of the dose of antihistamines (benadryl 40 mg), and subsequent infusions without allergic reactions did not require the use of nonsteroidal anti‐inflammatory drugs. Sixty‐eight patients were included in the study in the routine infusion group, with 121 infusions in total, and 129 patients were included in the rapid infusion group, with a total of 738 infusions (multiple infusions were recorded for the same patient), of which 59 infusions were given in the third round, 304 infusions in the fourth–ninth rounds, and 375 infusions in the 10 or higher rounds.

**TABLE 2:** Daratumumab regimen and frequency.

Characteristics	*N* = 129	*F* = 859
Daratumumab regimen		
Dara‐Vrd	85 (65.89%)	498 (57.97%)
Dara‐Vd	20 (15.51%)	204 (23.75%)
Dara‐Pd	8 (6.2%)	52 (6.05%)
Dara‐Kd	10 (7.75%)	70 (8.15%)
Other	6 (4.65%)	35 (4.08%)
Previous lines of therapy, *n*		
Median (range)	(1–6)	
1	105 (81.4%)	/
2–3	18 (13.95%)	/
>3	6 (4.65%)	/
Pre‐dose		
Celecoxib 200 mg, Diphenhydramine 40 mg, methylprednisolone 40 mg	68 (52.71%)	93 (10.83%)
Diphenhydramine 20 mg, dexamethasone 40 mg	129	766 (89.17%)
Routine infusions		
First dose	68 (52.71%)	68 (7.91%)
Second dose	53 (41.09%)	53 (6.17%)
Rapid daratumumab infusions		
Third dose	59 (45.74)	59 (6.87%)
Fourth–ninth dose	74 (57.36%)	304 (35.39%)
≥Tenth dose	65 (50.39%)	375 (43.66%)

Abbreviations: D, dexamethasone; *F*, frequency; K. carfilzomib; *N*, number; P, pomalidomide; r, lenalidomide; V, bortezomib.

### 3.2 Complications of routine infusion versus rapid infusion

Our findings showed (Table 3) that there was no difference in age or sex between the conventional infusion group and the rapid infusion group (*p* > 0.05), nor was there a difference in age stratification (≥60 vs. <60 years) (*p* = 0.218). The incidence of IRRs was 27.27% in the conventional infusion group and 1.35% in the rapid infusion group, and there was also a difference in the symptoms associated with IRRs between the two groups (*p* < 0.001). Only one patient in the rapid infusion group permanently stopped DARA infusion due to the occurrence of tertiary IRRs, and the remaining nine infusions were completed successfully after the use of antihistamines. There was also a difference in the time to infusion after the occurrence of an infusion reaction between the two groups (*p* < 0.001).

**TABLE 3:** Comparison of infusion‐related reactions and general information in different daratumumab infusion regimens.

Characteristics	Routine infusions		Rapid daratumumab infusions		*χ* ^*2*^/*F*	*p*‐Values
Age (years)						
≥60	28 (41.18%)	/	65 (50.39%)	/	1.516	0.218
<60	40 (58.82%)	/	64 (49.61%)	/		
Sex, *n* (%)						
Male	35 (51.47%)	/	73 (56.59%)	/	0.471	0.493
Female	33 (48.53%)	/	56 (43.41%)	/		
Underlying disease						
Yes	10 (14.71%)	/	25 (19.38%)	/	0.666	0.415
No	58 (85.29%)	/	104 (80.62%)	/		
Grade	*N* = 68	*F* = 121	*N* = 129	*F* = 738		
0	36 (52.94%)	88 (72.73%)	121 (93.8%)	728 (98.65%)	50.079	<0.001
1	9 (13.24%)	9 (7.44%)	5 (3.88%)	6 (0.81%)		
2	21 (30.88%)	21 (17.35%)	3 (2.32%)	4 (0.54%)		
3	3 (4.41%)	3 (2.48%)	0	0		
Symptoms	*N* = 33	*F* = 33	*N* = 7	*F* = 10		
Respiratory symptoms only	10 (30.3%)	10 (30.3%)	1 (14.3%)	2 (20%)	40	<0.001
Respiratory and cardiovascular symptoms	13 (39.39%)	13 (39.39%)	2 (28.6%)	3 (30%)		
Respiratory and cutaneous symptoms	6 (18.18%)	6 (18.18%)	2 (28.6%)	3 (30%)		
Other symptoms (gastrointestinal, general)	4 (12.12%)	4 (12.12%)	2 (28.6%)	2 (20%)		
Outcome	/	/	N = 7	F = 10	/	/
Rapid daratumumab infusion continued	/	/	3 (42.85%)	3 (30%)		
Converted to routine infusion	/	/	3 (42.85%)	5 (60%)		
Permanent daratumumab discontinuation	/	/	1 (14.3%)	1 (30%)		
Average infusions time after IRRs occurred($\bar{x}$ ± *SD*, range)	11.21 ± 4.26 (6.5–22)		7.25 ± 5.08 (1.5–21)		24.781	<0.001

Abbreviations: AEs, adverse events; *F*, *F* statistical values; F, frequency; IRRs, infusion‐related reactions; *N*, number; *χ* ^*2*^, Chi‐square test values.

### 3.3 Comparison of rapid infusion results across studies

As shown in Table 4, there were nine studies on the safety of rapid infusion of DARA, including seven studies on the safety of 90‐min rapid infusion of DARA, one study on the safety of infusion for no more than 110 min, and one study on the safety of 120‐min rapid infusion. The number of cases in each study ranged from 25 to 147, and the incidence of IRRs was <6%, with the highest grade of IRRs occurring being grade 3.

**TABLE 4:** Summary of data from studies of rapid infusion of daratumumab.

Study	Year	Type	Include patients	Infusion programme	IRR/AEs^a^ occurrence with Rapid infusions
Barr et al. [9]	2018	Prospective, single center	28	90 min rapid infusion	No IRRs 1 (3.5%) occurred grade 2 hypertension event
Hamadeh et al. [10]	2020	Retrospective, single center	53	90 min rapid infusion	1 (1.9%) occurred IRRs after Day 8 of Cycle 1; 1 (5.5%, *n* = 18) occurred IRRs after Day 15 of Cycle 1
Gozzetti et al. [11]	2020	Prospective, single center	39	90 min rapid infusion	No IRRs
Lombardi et al. [12]	2021	Retrospective, single center	25	90 min rapid infusion	4 (8%) occurred grade 1 IRRs
Patel et al. [13]	2021	Retrospective, single center	75	90 min rapid infusion	1 (103 infusions, 0.97%) infusion occurred grade 1 in IRRs.
Gordan et al. [14]	2021	Retrospective, single center	147	Rapid infusion <110 min, after >2 infusions	No IRRs
Bonello et al. [15]	2022	Retrospective, multicenter	134	90 min rapid infusion	7 (5%) occurred grade 1 and 2 IRRs.
Stakiw et al. [16]	2023	Prospective, multicenter	40	90 min rapid infusion	No occurred grade 3 IRRs
Wang et al. [17]	2023	Prospective, single center	60	120 min: 20% of the dose in 30 min (200 mL/h), 80% in 60 min (270 mL/h), after >2 infusions	3 (5%) occurred grade 1 and 2 IRRs.

Abbreviations: AEs, adverse events; IRRs, infusion‐related reactions.

^a^ Occurred during the rapid infusion.

## 4 DISCUSSION

Although DARA was approved by the US Food and Drug Administration (FDA) in 2015, it was only approved for marketing in China in 2019, and with its official inclusion in the Chinese National Basic Medical Insurance, Workers' Compensation Insurance and Maternity Insurance Drug Catalog (2021)^18^ in 2021, there has been a surge in demand for drugs from patients. Like for other monoclonal antibodies, infusion‐related adverse reactions (IRRs), including upper respiratory symptoms (cough, throat irritation, nasal congestion, wheezing, or shortness of breath), chills, rash, and gastrointestinal symptoms, are the most common adverse events (AEs) in patients treated with DARA.^19^, ^20^

In our study, the incidence of IRRs was 27.27% in the standard infusion group and 1.35% in the rapid infusion group, which are comparable (*p* < 0.001). Our findings confirmed the results of the Barr et al.^9^ study, indicating the safety and feasibility of our regimen, which is consistent with the results of other studies.^9^, ^10^, ^11^, ^12^, ^13^, ^14^, ^15^, ^16^, ^17^ We found that accelerating the DARA infusion rate for subsequent infusions after the second infusion reduces the infusion time, and IRRs are less common.

For the first half hour of infusion, our protocol was consistent with that in other studies, but our infusion rate at the remaining 80% of the DARA dose was controlled at a maximum of 300 mL/h, mainly based on our national nursing infusion standards.^21^ The infusion drip rate for adults is generally no more than 100 drops/min, based on the infusion coefficient of the infuser of 20 drops/ml, which translates to no more than 300 mL/h. Compared to 90 min of rapid infusion, although the infusion of the remaining 80% of the DARA dose was slower in our study, the incidence of IRRs was also reduced compared to that in the study of Wang et al.,^17^ which showed an increase in the infusion rate, a reduction in the infusion time, and a reduction in the incidence of IRRs, similar to the results of Gorden et al.^14^

We also compared the mean infusion time after the occurrence of IRRs in the standard infusion and rapid infusion groups, and the mean infusion time after the occurrence of IRRs in the patients in the rapid infusion group was significantly less than that in the standard infusion group (*p* < 0.001), indicating that the rapid infusion group could also end the infusion smoothly after timely treatment of IRRs, which still greatly shortened the infusion time. In addition, we found that age (≥60 or <60 years) had no effect on the choice of DARA infusion or the method of infusion (*p* = 0.218), suggesting that older patients can also tolerate the rapid drip regimen. Sex and underlying disease also did not affect the choice of infusion method in this study.

According to the results from the rapid titration group, no patient had IRRs above grade 3; only one patient experienced interruption of the DARA infusion after the occurrence of IRRs, and there was no subsequent DARA infusion because of this patient's relatively old age (77 years), underlying hypertension, and physical intolerance. The remaining patients who experienced IRRs were able to complete the infusion successfully after treatment with antihistamines. For reinfusion of DARA in patients who had not been infused with DARA for a long period, referring to the Bonello et al. study,^9^ we set the time to interrupt the infusion for a period of no more than 2 months, which makes it safer to reinitiate the regular infusion for an interruption time of more than 2 months, compared to the 6 months recommended by Hamadeh et al.^10^ Two patients in this study had interruptions in their DARA infusions for more than 2 months (one for 7 months and one for 3 months), and no IRRs occurred with the standard infusion regimen or again with the rapid infusion regimen.

In a comprehensive comparison of all other rapid infusion literature,^9^, ^10^, ^11^, ^12^, ^13^, ^14^, ^15^, ^16^, ^17^ our study protocol showed a low rate of adverse events and low severity of IRRs with 738 rapid DARA infusions. Hamadeh et al.^10^ developed a model for analyzing 53 patients who received DARA infusions. Patients with rapid DARA infusions outperformed patients with standard infusions after 53 weeks of injections by saving $1.5w. Our study also demonstrated for the first time that our rapid‐infusion DARA regimen is suitable for Chinese MM patients and can significantly shorten the infusion time, optimize the length of hospital stay, indirectly reduce caregiver labor costs, and increase patient comfort.

Currently, there are studies being conducted on other ways of using DARA, such as IV formulations and subcutaneous (SC) formulations,^21^, ^22^, ^23^, ^24^ but they are still in the clinical trial stage, their popularity is not increasing rapidly, and they will take time to enter China. We believe that in the next few years, more new methods of using DARA will be applied in the clinic, which will significantly shorten the infusion time and not only will be safe for patients but also will be effective in reducing the infusion time. This, in turn, will reduce the burden on clinical resources and the financial costs, as well as improve the patient experience.

## AUTHOR CONTRIBUTIONS

**Xi‐xi Yin:** Writing – review and editing (lead). **Yueyun Hu:** Data curation (equal). **Yusi Yang:** Data curation (equal). **Xinglan Zhang:** Project administration (equal). **Li Liu:** Project administration (equal). **Xi Cao:** Project administration (equal). **Jianwen Chen:** Writing – review and editing (equal). **Zhongjun Xia:** Project administration (equal). **Ye Wang:** Writing – review and editing (lead).

## CONFLICT OF INTEREST STATEMENT

The authors declare no conflicts of interest.

## Data Availability

Data available on request due to privacy/ethical restrictions. The data that support the findings of this study are available on request from the corresponding author. The data are not publicly available due to privacy or ethical restrictions.
